# Influence of Local Anesthesia Techniques on the Microstructural Integrity of Dental Injection Needles: A Field Emission Scanning Electron Microscopy Analysis

**DOI:** 10.3390/bioengineering13091087

**Published:** 2026-09-19

**Authors:** Fian Dervis, Alp Saruhanoglu, Duygu Ofluoglu, Meltem Koray

**Affiliations:** 1 Institute of Graduate Studies in Health Sciences, Istanbul University, 34126 Istanbul, Türkiye; fiandervis@gmail.com; 2 Department of Oral and Maxillofacial Surgery, Faculty of Dentistry, Istanbul University, 34126 Istanbul, Türkiye; duyguofluoglu@gmail.com (D.O.); mkoray@istanbul.edu.tr (M.K.)

**Keywords:** dental needles, local anesthesia, field emission scanning electron microscopy, needle deformation, inferior alveolar nerve block

## Abstract

Dental injection needle integrity may influence tissue trauma during local anesthesia. This study compared needle-tip microstructural deformation after different local anesthesia techniques using field emission scanning electron microscopy (FESEM). A total of 110 single-use needles were allocated to an unused descriptive control set (*n* = 5), infiltrative local anesthesia (ILA; *n* = 35), direct inferior alveolar nerve block anesthesia (DIANBA; *n* = 35), and indirect inferior alveolar nerve block anesthesia (IIANBA; *n* = 35). After one clinical application, tips were evaluated for bevel edge irregularity (BEI), tip axis deviation (TAD), and bevel edge microstructural breakdown (BEMB). Inferential analyses were restricted to the three equally sized clinical groups. BEI occurred in 5.7%, 28.6%, and 45.7% of the ILA, DIANBA, and IIANBA groups, respectively (overall *p* = 0.0007; Cramer’s V = 0.371). After Holm adjustment, BEI differed between ILA and DIANBA (adjusted *p* = 0.0469) and between ILA and IIANBA (adjusted *p* = 0.0007). TAD frequencies were 14.3%, 34.3%, and 40.0% (overall *p* = 0.0466; V = 0.242), but no pairwise comparison remained significant after adjustment. BEMB frequencies were 22.9%, 40.0%, and 45.7%, with no statistically significant overall difference (*p* = 0.117). Thus, the demonstrated finding is greater needle-tip microstructural deformation, particularly BEI, after nerve block techniques; clinical consequences such as pain or tissue trauma were not assessed and require dedicated investigation.

## 1. Introduction

Local anesthesia (LA) is an indispensable component of contemporary dental practice, enabling a wide range of diagnostic and therapeutic procedures to be performed with minimal pain and discomfort. Despite its routine use, the injection itself remains one of the most common causes of dental anxiety and is frequently associated with pain, discomfort, and treatment avoidance. Pain experienced during LA administration is multifactorial and is influenced by several variables, including injection technique, injection speed, tissue characteristics, anesthetic solution properties, and the mechanical characteristics of the injection needle [1,2,3,4,5,6].

Among these factors, the structural integrity of the dental injection needle has received increasing attention because it may directly influence tissue trauma during needle penetration. Previous studies have demonstrated that even a single clinical use may induce microstructural alterations, including bevel edge irregularities, tip deformation, hook formation, and axial deviation, particularly following contact with cortical bone during inferior alveolar nerve block procedures [7,8,9,10,11,12,13,14,15]. Such alterations may increase tissue resistance during insertion and withdrawal, potentially resulting in greater tissue injury and patient discomfort.

Scanning electron microscopy (SEM) has been widely used to evaluate the morphology of dental needles after clinical use [7,8,9,10,11,12,13,14,15]. However, conventional SEM may have limited sensitivity for detecting subtle surface alterations at the submicron level. Field emission scanning electron microscopy (FESEM) provides substantially higher spatial resolution, greater depth of field, and improved image quality through the use of a field-emission electron source, enabling more detailed characterization of surface morphology and early microstructural damage. In addition, the high beam brightness and low voltage imaging capability of FESEM allow conductive materials such as stainless-steel needles to be examined without the application of conductive surface coatings, thereby preserving the native surface morphology and minimizing preparation-induced artifacts [16,17,18,19,20]. Although FESEM has been extensively applied in the evaluation of dental biomaterials and hard tissues, including implant surfaces, restorative materials, and root canal sealers [21,22,23,24,25,26,27,28], its application in the assessment of deformation of dental injection needles remains limited.

Dental needles are engineered as thin, sharp stainless-steel instruments whose cutting performance depends on the continuity, symmetry, and alignment of the bevel. During penetration, axial compression acts along the shaft, whereas contact with dense tissue or bone can introduce lateral and bending forces at the tip. A linear insertion is therefore mechanically different from an insertion that includes bone contact followed by redirection. In the latter situation, the bevel may be exposed to combined bending and torsional loading while it remains constrained within tissue. Experimental work on needle deflection and bevel design supports the premise that insertion path, bevel geometry, and contact conditions can alter needle behavior [7,12,14,15]. These considerations make the injection technique a clinically relevant exposure when comparing post-use needle morphology.

Existing studies have generally described deformation after infiltration or inferior alveolar nerve block, but direct comparisons of infiltrative, direct block, and indirect block techniques under standardized imaging conditions remain limited. Moreover, morphology and clinical effect should not be treated as interchangeable endpoints. A microscopic defect can be demonstrated by imaging, whereas any consequence for pain, bleeding, soft-tissue injury, postoperative discomfort, or needle handling requires patient- or clinician-level measurements. The present study was designed to address the morphological question only and to provide effect estimates that can inform appropriately powered clinical investigations.

Therefore, the present study aimed to evaluate and compare the microstructural deformation of dental injection needle tips following different local anesthesia techniques using FESEM. We hypothesized that techniques involving greater mechanical stress and needle redirection, particularly inferior alveolar nerve block techniques, would result in more pronounced microstructural damage than infiltrative anesthesia.

## 2. Materials and Methods

### 2.1. Study Design

This comparative experimental study was approved by the ethics committee (No: 09.2023.618) and performed at Istanbul University Faculty of Dentistry Department of Oral and Maxillofacial Surgery. All procedures were performed in accordance with the ethical standards of the institutional research committee and the principles of the Declaration of Helsinki. Written informed consent was obtained from all participants before inclusion in the study.

An a priori sample size calculation was performed using G*Power version 3.1 (Heinrich Heine University Düsseldorf, Germany) for an omnibus chi-square comparison among the three clinical groups. Based on the magnitude of the between-group difference reported by Dau et al. [7], the calculation used a Cohen effect size of w = 0.39, two degrees of freedom, a significance level of α = 0.05, and 95% power (1 − β = 0.95). The calculated total was 102 needles; this was rounded upward to 105 to provide equal groups of 35. Accordingly, 105 single-use stainless-steel needles (SET^®^ Inject, 27G × 1½, 0.40 × 40 mm; SET Medical, Istanbul, Türkiye) were included in the ILA, DIANBA, and IIANBA groups. Five additional unused needles from the same product were examined only as a descriptive morphological baseline to verify the appearance of unused bevels and to identify obvious pre-use defects. They were not intended to estimate control-group variance and were excluded from all inferential comparisons; therefore, their smaller number does not create an unbalanced inferential group.

### 2.2. Clinical Procedure

All local anesthesia procedures were performed by a single experienced oral and maxillofacial surgeon to ensure procedural standardization and eliminate operator-related variables. Each needle was used for a single tissue penetration and injection to ensure that the observed microstructural changes reflected the effects of only one clinical application.

For ILA, the needle was inserted into the vestibular mucobuccal fold using a conventional technique while avoiding intentional contact with bone. In the DIANBA, the needle followed a linear path until bone contact was established at the medial ramus of the mandible. The IIANBA technique involved a primary bone contact followed by a change in the needle axis while still within the tissue to bypass the lingula mandibulae.

Immediately after the injections, each needle was carefully removed by the hub to avoid secondary mechanical deformation. The sterilization procedure was performed solely for biosafety before FESEM examination and was applied identically to all specimens, including the unused control needles.

### 2.3. FESEM Analysis

Needles were analyzed at the Central Research Laboratory of Yıldız Technical University using a Thermo Scientific Apreo 2 S Field Emission Scanning Electron Microscope (FE-SEM; Thermo Fisher Scientific, Waltham, MA, USA). No conductive coating was applied prior to imaging to preserve the original surface characteristics.

FESEM imaging was performed using an Everhart-Thornley Detector (ETD; Thermo Fisher Scientific, Waltham, MA, USA) in standard imaging mode at an accelerating voltage of 2.0 kV. Images were acquired at ×160 magnification with a horizontal field width (HFW) of 2.59 mm and a working distance ranging from 24.7 to 26.2 mm. A 500 µm scale bar was used for all representative micrographs. All specimens were examined under identical imaging conditions and evaluated at predetermined magnifications according to study protocol criteria (Table 1) established before image assessment. The evaluation was performed independently by two blinded oral and maxillofacial surgeons using identical image magnifications and direct comparison with unused control needles. Each feature was recorded as present or absent only when the predefined criteria were fulfilled. Any discrepancies were resolved by consensus. Disagreements occurred in only two specimens (both involving BEI assessment) and were resolved by consensus before the final analysis. The consensus assessment was used for final statistical analyses. The control group served as the reference for the evaluation of all microstructural changes.

The assessment unit was the individual needle. The observers reviewed the standardized images without access to the clinical group label. The three outcomes represented distinct morphological features and were not combined into a composite score. Representative micrographs are presented for visual illustration; frequency estimates and statistical analyses were based on the blinded assessment of the complete set of 110 needles, not on the displayed examples alone.

Because the outcomes were binary, a specimen could be positive for more than one feature; the denominator for each outcome remained the number of needles in that group. The observers assessed BEI, TAD, and BEMB separately before any group-level frequencies were calculated. This sequence prevented the observed group distribution from being used to resolve an individual rating. Initial agreement was summarized descriptively, and consensus ratings were used only after the independent assessments had been completed.

- Bevel Edge Irregularity (BEI) was defined as the presence of one or more localized interruptions of the normally smooth cutting edge, including notching, nicking, or loss of edge continuity, compared with unused control needles.
- Tip Axis Deviation (TAD) was defined as any permanent bending or deviation of the needle tip from its original longitudinal axis relative to unused control needles. Minor optical distortion caused by image orientation was not considered TAD.
- Bevel Edge Microstructural Breakdown (BEMB) was defined as localized disruption of the bevel surface characterized by microcracks, fractures, chipping, or surface collapse affecting the integrity of the cutting edge.

### 2.4. Statistical Analysis

Statistical analyses were performed using IBM SPSS Statistics for Windows, version 27.0 (IBM Corp., Armonk, NY, USA). The unused needles were treated as a descriptive morphological baseline and were excluded from hypothesis testing. For each binary FESEM outcome, ILA, DIANBA, and IIANBA were first compared using a 2 × 3 Pearson chi-square test. Cramer’s V was reported as the omnibus effect size. Irrespective of the omnibus result, all three prespecified pairwise comparisons were then performed with two-sided Fisher’s exact tests to provide direct technique-to-technique comparisons. The three pairwise *p*-values within each outcome were adjusted by the Holm step-down procedure to control the family-wise error rate. Conditional maximum-likelihood odds ratios (ORs) with exact 95% confidence intervals (CIs) were reported as pairwise effect estimates; CIs were not multiplicity-adjusted. Group proportions were accompanied by Wilson 95% CIs. Statistical significance was defined as an adjusted two-sided *p*-value < 0.05.

## 3. Results

**Figure 1:**
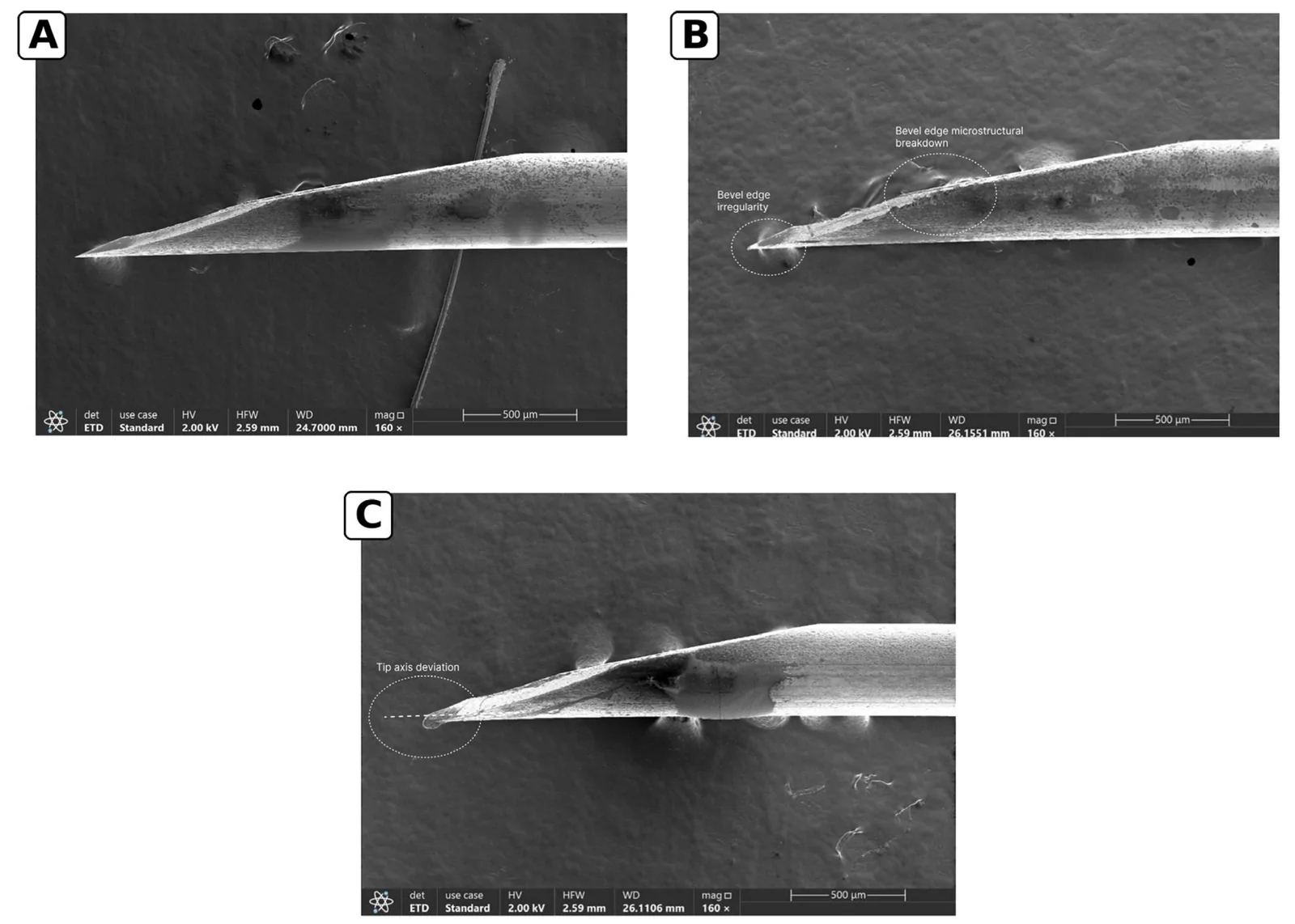
Representative FESEM micrographs presented as a consolidated multipanel figure: (**A**) an unused control needle with a smooth surface and sharp bevel edge; (**B**) a needle after DIANBA showing BEI and BEMB; and (**C**) a needle after IIANBA showing TAD. Images were acquired at 160× with a 500 µm scale bar. The panels illustrate the scoring criteria and do not represent a severity scale.

FESEM examination of all five unused needles demonstrated sharp bevel edges without BEI, TAD, or BEMB. The unused specimens were used only to illustrate baseline morphology and were not included in inferential analyses. Among the clinical groups, the ILA specimens showed relatively few bevel-edge defects, whereas DIANBA and IIANBA specimens showed more frequent irregularity and deviation. Figure 1 consolidates the available standardized representative micrographs into a single multipanel figure. The panels are illustrative examples of the predefined outcomes; all 110 needles, rather than only the displayed examples, contributed to the blinded morphological assessment.

BEI was observed in 2 of 35 ILA needles (5.7%; 95% CI, 1.6–18.6%), 10 of 35 DIANBA needles (28.6%; 95% CI, 16.3–45.1%), and 16 of 35 IIANBA needles (45.7%; 95% CI, 30.5–61.8%) (Table 2). The difference among the three clinical groups was significant (χ^2^(2) = 14.42, *p* = 0.0007; Cramer’s V = 0.371). In the direct comparisons, DIANBA had higher odds of BEI than ILA (OR = 6.44; 95% CI, 1.21–65.58; Holm-adjusted *p* = 0.0469), and IIANBA had higher odds than ILA (OR = 13.38; 95% CI, 2.70–132.52; adjusted *p* = 0.0007). IIANBA did not differ significantly from DIANBA (OR = 2.08; 95% CI, 0.70–6.42; adjusted *p* = 0.2159) (Table 3 and Table 4).

TAD was detected in 5 of 35 ILA needles (14.3%; 95% CI, 6.3–29.4%), 12 of 35 DIANBA needles (34.3%; 95% CI, 20.8–50.8%), and 14 of 35 IIANBA needles (40.0%; 95% CI, 25.6–56.4%). The omnibus comparison was significant at the 0.05 level (χ^2^(2) = 6.13, *p* = 0.0466; Cramer’s V = 0.242). However, none of the three direct comparisons remained significant after Holm adjustment: DIANBA versus ILA, adjusted *p* = 0.1856; IIANBA versus ILA, adjusted *p* = 0.0900; and IIANBA versus DIANBA, adjusted *p* = 0.8049. The unadjusted IIANBA-versus-ILA Fisher *p*-value was 0.0300, showing why adjustment for the prespecified family of comparisons changes the inference.

BEMB was identified in 8 of 35 ILA needles (22.9%; 95% CI, 12.1–39.0%), 14 of 35 DIANBA needles (40.0%; 95% CI, 25.6–56.4%), and 16 of 35 IIANBA needles (45.7%; 95% CI, 30.5–61.8%). BEMB proportions were numerically higher in the two nerve block groups, although this difference was not statistically significant (χ^2^(2) = 4.29, *p* = 0.117; Cramer’s V = 0.202). No pairwise comparison was significant after Holm adjustment (all adjusted *p* ≥ 0.231). These data are therefore reported as a descriptive pattern rather than evidence of a between-technique difference.

Initial observer agreement was 108 of 110 needles (98.2%) for BEI and 110 of 110 (100%) for both TAD and BEMB. The two BEI disagreements were reviewed jointly and resolved by consensus before group frequencies were tabulated. Thus, the reported counts reflect the final consensus dataset while the descriptive agreement values reflect the initial independent readings.

## 4. Discussion

The principal demonstrated finding was a technique-related difference in FESEM-detected needle-tip morphology. BEI showed the clearest association: both inferior alveolar nerve block groups had significantly higher odds than ILA after family-wise adjustment, with a moderate omnibus effect (Cramer’s V = 0.371). TAD showed a weaker omnibus association (V = 0.242), and its initially suggestive pairwise pattern did not remain significant after Holm adjustment. BEMB also occurred numerically more often after the nerve block techniques, but neither the omnibus nor adjusted pairwise tests supported a statistically significant difference. The revised interpretation therefore focuses on BEI and does not generalize statistical significance to every deformation outcome.

Previous SEM-based studies have consistently shown that dental needles undergo deformation after clinical use. Rout et al. [8] reported deformation in 97.3% of 27-gauge needles following bone contact during inferior alveolar nerve block administration, while Almendros-Marqués et al. [9] observed bevel edge deformation after a single clinical use. Similarly, Skapetis et al. [10] described bevel notching, hook formation, and tip axis deviation following inferior alveolar nerve block injections. The present findings are consistent with these observations and further demonstrate that the severity of deformation increases according to the complexity of the injection technique. In particular, the indirect inferior alveolar nerve block technique resulted in the highest frequencies of bevel edge irregularity and tip axis deviation, suggesting that repeated changes in needle direction within the tissues generate additional torsional and bending stresses.

The gradation in BEI frequencies is mechanically plausible. ILA was performed without intentional bone contact, whereas both inferior alveolar nerve block techniques required advancement to a bony landmark. The indirect technique also required alteration of the needle axis while the needle remained within tissue. Bone contact can load the bevel asymmetrically, while redirection can add bending and torsional components. The present observational morphology cannot isolate the contribution of each force, but the standardized needle product and single operator reduce two important alternative sources of variability. The lack of a significant DIANBA-IIANBA difference also means that the data do not establish redirection itself as an independent cause; that mechanism should be tested in a controlled bench model that measures contact angle, insertion force, and number of directional changes.

Needle geometry and manufacturing characteristics have also been reported to influence deformation behavior. Gaitan-Fonseca et al. [11] demonstrated that the extent of bevel deformation varies among commercially available dental needles. To minimize this potential source of bias, all procedures in the present study were performed using needles of identical gauge and from the same manufacturer, allowing the local anesthesia technique to be evaluated as the principal experimental variable.

Unlike previous investigations that primarily relied on conventional scanning electron microscopy, the present study employed FESEM for microstructural evaluation. The higher spatial resolution, greater depth of field, and improved image quality provided by FESEM enabled detailed visualization of subtle surface alterations that may not be readily detected using conventional SEM [16,17,18,19,20,29]. In addition, the stainless-steel needles were examined without conductive surface coating, thereby preserving their native surface morphology and eliminating coating-related artifacts. This methodological approach increased confidence that the observed defects reflected true clinical deformation rather than specimen preparation artifacts.

Although FESEM has been widely used to characterize dental biomaterials, implant surfaces, restorative materials, and endodontic materials [21,22,23,24,25,26,27,28], its application to the evaluation of dental injection needles remains limited. To our knowledge, this is one of the first comparative studies to investigate needle tip deformation following different local anesthesia techniques using FESEM. The findings therefore extend the existing literature by providing high-resolution evidence that the magnitude of microstructural damage is influenced by the mechanical characteristics of the local anesthetic technique itself.

Reporting effect estimates adds context beyond dichotomous significance. The wide exact CIs around the pairwise ORs, particularly for comparisons involving the low BEI count in ILA, indicate limited precision despite the significant adjusted tests. For TAD and BEMB, the intervals include both small and potentially important effects, so absence of statistical significance should not be interpreted as proof of equivalence. Larger studies can use the observed proportions and CIs to plan confirmatory comparisons, ideally with a prespecified primary morphological outcome and fewer multiplicity concerns.

The three morphological endpoints should also be interpreted separately. BEI captures disruption of the cutting edge, TAD captures a change in tip alignment, and BEMB captures localized fracture-like surface loss. A needle may therefore meet more than one criterion, and the present binary ratings do not establish an ordinal sequence from minor to severe damage. For the same reason, the representative panels in Figure 1 illustrate recognizable features but should not be read as a calibrated severity series. A future severity framework should be defined before image review and supported by quantitative measures such as angular deviation, defect length or area, and surface-roughness parameters. Such measurements would improve discrimination within each category and allow assessment of dose-response relationships with bone contact and redirection.

The unused specimens addressed a different question from the clinical groups. Their role was to confirm the sampled baseline appearance of an unused needle of the same product, not to estimate the full distribution of manufacturing defects. Reclassifying them as a descriptive reference avoids an unstable inferential comparison based on five observations and aligns the statistical analysis with the a priori allocation of 35 needles to each clinical technique. Nevertheless, baseline manufacturing variation remains relevant because a post-use defect cannot always be attributed uniquely to clinical loading without pre-use imaging of the same item. A definitive design could combine a larger, lot-stratified unused sample with non-destructive pre-use imaging or matched production controls, while maintaining sterility and blinding. This would help separate pre-existing surface variation from deformation acquired during the injection.

The study also has design features that support internal consistency. The three clinical groups were balanced, the same needle gauge and manufacturer were used throughout, one experienced operator performed all injections, and the imaging settings and binary definitions were standardized before assessment. Independent blinded review produced high initial agreement. These features reduce variation arising from product, operator, imaging, and classification differences, although they do not remove the limitations of a single-center study or substitute for quantitative clinical endpoints.

The findings do not, by themselves, justify a clinical recommendation to replace a needle after every injection. The study demonstrates post-use microstructural deformation but did not measure whether that deformation was felt by patients, produced visible tissue injury, altered penetration or withdrawal force, or affected anesthetic success. Permanent nerve involvement has been reported following inferior alveolar nerve block injections [30]; however, the present study did not assess neurosensory outcomes and cannot establish any relationship between needle-tip deformation and nerve injury. Clinicians should continue to follow manufacturer instructions, single-use requirements, and established safe injection practice. If resistance, visible damage, or a need for substantial redirection raises concern about needle integrity, replacement may be considered according to clinical judgment; this statement is a cautious practical consideration rather than an outcome demonstrated by the present data. Prospective studies linking FESEM findings to patient-reported pain and objective clinical endpoints are needed before a routine replacement interval can be recommended.

Several limitations should be acknowledged. First, the investigation was designed around a morphological endpoint and did not directly assess patient-reported injection pain, intraoperative or postoperative discomfort, bleeding, soft-tissue trauma, anesthetic success, withdrawal force, or clinician-observed needle performance. Consequently, no causal or quantitative link can be drawn between the FESEM findings and clinical harm or patient experience. Second, the five unused needles constituted a descriptive baseline rather than a powered comparison group. This design can document the appearance of the sampled unused needles but cannot precisely characterize manufacturing variance. Third, only 27-gauge stainless-steel needles from one manufacturer were studied, limiting generalizability to other gauges, bevel geometries, alloys, and production lots. Fourth, the binary scoring system improves reproducibility but does not grade defect severity or quantify surface area, displacement, or roughness. Fifth, all procedures were performed by one experienced surgeon; this standardizes technique but does not capture interoperator variation. Finally, the pairwise ORs have wide CIs, and multiple testing reduced the strength of the TAD evidence. Future work should enlarge the unused reference sample, incorporate quantitative three-dimensional profilometry or mechanical testing, prespecify severity measures, and collect patient- and clinician-reported outcomes.

## 5. Conclusions

Within the limits of this study, local anesthesia technique was associated with FESEM-detected microstructural deformation of dental injection needles. The most robust finding was a higher frequency of bevel edge irregularity after direct and indirect inferior alveolar nerve block anesthesia than after infiltrative anesthesia. Evidence for tip axis deviation was weaker after adjustment for multiple pairwise testing, and bevel edge microstructural breakdown did not differ significantly among the three clinical groups. These results demonstrate morphological change only. They do not establish effects on injection pain, tissue trauma, bleeding, postoperative discomfort, or needle performance. Studies that measure these clinical outcomes alongside quantitative needle deformation are required before practice recommendations can be made.

## Figures and Tables

**Table 1:** FESEM-based criteria used for the evaluation of dental needle-tip deformation.

Parameter	Definition/Representative FESEM Findings
BEI	Notching, nicking, flattening, or loss of bevel edge continuity
TAD	Deviation of the needle tip from its original longitudinal axis, with or without tip bending
BEMB	Microcracks, chipping, fracture lines, localized surface collapse, or material loss

**Table 2:** Distribution of FESEM-detected needle-tip deformation according to local anesthesia technique.

Group	*n*	BEI, *n* (%) [95% CI]	TAD, *n* (%) [95% CI]	BEMB, *n* (%) [95% CI]
Unused baseline *	5	0	0	0
ILA	35	2 (5.7) [1.6–18.6]	5 (14.3) [6.3–29.4]	8 (22.9) [12.1–39.0]
DIANBA	35	10 (28.6) [16.3–45.1]	12 (34.3) [20.8–50.8]	14 (40.0) [25.6–56.4]
IIANBA	35	16 (45.7) [30.5–61.8]	14 (40.0) [25.6–56.4]	16 (45.7) [30.5–61.8]

Values for clinical groups are *n* (%) with Wilson 95% confidence intervals. * The five unused needles were a descriptive morphological baseline and were excluded from inferential analyses. Abbreviations: BEI, bevel edge irregularity; TAD, tip axis deviation; BEMB, bevel edge microstructural breakdown; ILA, infiltrative local anesthesia; DIANBA, direct inferior alveolar nerve block anesthesia; IIANBA, indirect inferior alveolar nerve block anesthesia.

**Table 3:** Omnibus comparisons among the three clinical groups and effect sizes.

Outcome	χ^2^ (df = 2)	Overall *p*	Cramer’s V	Adjusted Pairwise Inference
BEI	14.42	0.0007	0.371	ILA-DIANBA and ILA-IIANBA significant
TAD	6.13	0.0466	0.242	None significant after Holm adjustment
BEMB	4.29	0.117	0.202	None significant

**Table 4:** Prespecified direct pairwise comparisons among clinical groups.

Outcome	Comparison	OR	Exact 95% CI	Raw *p*	Holm-Adjusted *p*
BEI	DIANBA vs. ILA	6.44	1.21–65.58	0.0234	0.0469
BEI	IIANBA vs. ILA	13.38	2.70–132.52	0.0002	0.0007
BEI	IIANBA vs. DIANBA	2.08	0.70–6.42	0.2159	0.2159
TAD	DIANBA vs. ILA	3.08	0.86–12.82	0.0928	0.1856
TAD	IIANBA vs. ILA	3.92	1.12–16.12	0.0300	0.0900
TAD	IIANBA vs. DIANBA	1.27	0.43–3.78	0.8049	0.8049
BEMB	DIANBA vs. ILA	2.22	0.71–7.37	0.1975	0.3950
BEMB	IIANBA vs. ILA	2.80	0.91–9.23	0.0769	0.2308
BEMB	IIANBA vs. DIANBA	1.26	0.44–3.63	0.8094	0.8094

ORs are conditional maximum-likelihood estimates; exact 95% CIs are unadjusted. Holm adjustment was applied separately to the three comparisons within each outcome.

## Data Availability

The raw data supporting the conclusions of this article will be made available by the authors on request.
